# An *In Vitro* System for Studying Murid Herpesvirus-4 Latency and Reactivation

**DOI:** 10.1371/journal.pone.0011080

**Published:** 2010-06-11

**Authors:** Janet S. May, Neil J. Bennett, Philip G. Stevenson

**Affiliations:** Division of Virology, Department of Pathology, University of Cambridge, Cambridge, United Kingdom; Karolinska Institutet, Sweden

## Abstract

The narrow species tropisms of Epstein-Barr Virus (EBV) and the Kaposi's Sarcoma -associated Herpesvirus (KSHV) have made Murid Herpesvirus-4 (MuHV-4) an important tool for understanding how gammaherpesviruses colonize their hosts. However, while MuHV-4 pathogenesis studies can assign a quantitative importance to individual genes, the complexity of *in vivo* infection can make the underlying mechanisms hard to discern. Furthermore, the lack of good *in vitro* MuHV-4 latency/reactivation systems with which to dissect mechanisms at the cellular level has made some parallels with EBV and KSHV hard to draw. Here we achieved control of the MuHV-4 lytic/latent switch *in vitro* by modifying the 5′ untranslated region of its major lytic transactivator gene, ORF50. We terminated normal ORF50 transcripts by inserting a polyadenylation signal and transcribed ORF50 instead from a down-stream, doxycycline-inducible promoter. In this way we could establish fibroblast clones that maintained latent MuHV-4 episomes without detectable lytic replication. Productive virus reactivation was then induced with doxycycline. We used this system to show that the MuHV-4 K3 gene plays a significant role in protecting reactivating cells against CD8^+^ T cell recognition.

## Introduction

Herpesvirus lifecycles are characterized by lytic infection, latency and reactivation. The known human gammaherpesviruses - Epstein-Barr virus (EBV) and the Kaposi's Sarcoma-associated Herpesvirus (KSHV) - have narrow species tropisms and show only limited lytic propagation *in vitro*. Consequently they are studied mainly as latent infections of transformed cells [1]. In contrast, Murid Herpesvirus-4 (MuHV-4), a close relative of KSHV [2], readily propagates by lytic replication such that tightly latent *in vitro* infections have proved hard to establish. MuHV-4 is typically studied *in vivo*. However, the complexity of pathogenesis studies makes difficult an understanding of the underlying mechanisms without additional experimental tools. Thus, while *in vitro* MuHV-4 lytic propagation has proved very useful for studying gammaherpesvirus lytic functions, the lack of *in vitro* MuHV-4 latency/reactivation systems has been a significant hindrance.

MuHV-4-infected NS0 myeloma cell cultures can be maintained over several weeks [3], but only because these cells are poorly infectible - their spontaneous reactivation rate is high and MuHV-4 lacking M7, which infects NS0 cells much more readily [4], cannot be maintained in this way. The S11 tumour cell line has been used to define some aspects of MuHV-4 latency [5] but again shows high reactivation rates and offers no way to compare recombinant viruses with the wild-type or uninfected controls. A subclone selected for lower reactivation rates proved to have an abnormal, integrated genome [6]. A predominantly latent infection of A20 B cells has been described [7]. However, A20 cells are very poorly infectible by MuHV-4 [8] and HCMV IE1 promoters such as that used to isolate infected clones are normally turned off in latency [9], [10]. Thus, the need for antibiotic selection may have biased the type of latency being studied. The lack of good *in vitro* latency systems has inevitably also made MuHV-4 reactivation hard to analyze. *Ex vivo* reactivation [11] is restricted to <1/1000 explanted cells even at peak virus loads, and generally requires further rounds of lytic replication for detection. Sub-cloned S11 cells [12] and antibiotic-selected A20 cells [7] can reactivate, but the proportion of reactivating cells was unclear and presumably low as there was no sign of reactivation terminating the infected cultures. In summary, the tools available to study MuHV-4 latency have been far from ideal.

It might be considered that analyzing MuHV-4 reactivation at all is unnecessary, since primary lytic replication occurs readily and has the same end-point. However, exogenous virions engage cellular receptors and deliver tegument proteins [13], while cells supporting reactivation may be conditioned by latent viral gene expression [14]. Therefore these processes are not necessarily the same. Without a good means of studying reactivation, some MuHV-4 gene functions have been hard to define. For example, immune evasion is presumed to play a key role in herpesvirus reactivation [15], but while MuHV-4 remains the only gammaherpesvirus for which CD8^+^ T cell evasion has been analyzed *in vivo* [16], the importance of this evasion for reactivation remains unknown. MuHV-4 lacking its K3 evasion gene [17]–[19] shows little defect in lytic replication, but rathers a CD8-dependent impairment of latency-associated lymphoproliferation. The increased *in vivo* presentation by K3^−^MuHV-4 of p79 [16], an epitope presented largely by B cells [20], suggests that K3 might also be important for reactivation, but it has not been possible to analyze the effect of K3 disruption on reactivation titers.

Comparing MuHV-4 primary lytic infection with reactivation is particularly difficult with B cells, since B cell infection remains poorly characterized. Early reports of phenotypic changes in splenic B cells exposed to MuHV-4 virions [21], [22] did not necessarily identify actual infection. Virion binding to B cells was subsequently shown to be poor [4] because this depends on heparan sulfate [23], which B cells do not express at high levels [24]. Further blocks are likely, as A20 B cells with artificially high level heparan sulfate expression remain poorly infectible [8]. Thus, in order to establish new tools for analyzing MuHV-4 reactivation we focussed on fibroblasts, for which primary MuHV-4 lytic infection is well-defined. Stromal cells are not a recognized site of EBV persistence [25], but MuHV-4 seems to be different to EBV in also establishing a persistent infection without B cells [26]. Thus, while B cells are the main site of MuHV-4 latency in lymphoid tissue [27] with a well-established role in virus transport [28], stromal infection may also play a fundamental role in the viral lifecycle [26], [29], [30]. Viral latency and reactivation in stromal cells may also be important in Kaposi's Sarcoma [31]. Thus, as a first step in understanding MuHV-4 latency/reactivation, we established an inducible infection of fibroblasts, and used this to define the importance of K3 in protecting reactivation against CD8^+^ T cell recognition.

## Materials and Methods

### Plasmids

pREV-TRE, pRevTet-On and pTet-tTS were from Clontech. The tetracycline-dependent transactivator (tTA) of pRevTet-On was subcloned into pREV-TRE by digesting pRevTet-On with *Bgl*I and pREV-TRE with *Bam*HI. Each was then blunted with T4 DNA polymerase and digested with *Cla*I. The blunt/*Cla*I tTA fragment was then ligated into pREV-TRE. A tetracycline-dependent transcriptional suppressor (tTS) was removed from pTet-tTS by digestion with *Eco*RI and *Cla*I and ligated into the *Eco*RI and *Cla*I sites of pSP73 (Promega Corporation). The retroviral expression vector pMSCV-IRES-PURO [18] was separately modified by cloning into its *Eco*RI/*Xho*I polylinker an *Eco*RI/*Xho*I fragment of the pSP73 polylinker, thereby gaining an additional *Bam*HI cloning site. The tTS fragment in pSP73 was then subcloned as an *Eco*RI/*Bgl*II fragment into the *Eco*RI/*Bam*HI sites of pMSCV-IRES-PURO.

### Viral mutagenesis

We generated a polyA-TRE construct by digesting pREV-TRE with *Xho*I and pSV40-ZEO2 (Invitrogen Corporation) with *Eco*RI. Both were then blunted with T4 DNA polymerase and digested with *Bam*HI, and the TRE fragment of pREV-TRE was ligated into pSV40-ZEO2 downstream of its SV40 polyadenylation site. The polyA-TRE construct was then amplified by PCR (Hi-Fidelity PCR kit, Roche Diagnostics Ltd.), including *Bsg*I recognition sites in the upstream and downstream primers, digested with *Bsg*I and cloned into a *Bsg*I site (genomic co-ordinate 66718) of a *Bam*HI-restricted MuHV-4 genomic clone (co-ordinates 64765–68813) in pUC19 [2]. The modified *Bam*HI genomic clone was then subcloned into the *Bam*HI site of the pST76K-SR shuttle vector and recombined into a MuHV-4 BAC by transient recA expression [32] to give TRE-50 MuHV-4. We derived a revertant BAC by recombining in the corresponding unmutated *Bam*HI clone. K3^−^TRE-50 MuHV-4 was generated by shuttling an established K3 mutation [10] onto the TRE-50 background. Viruses were reconstituted by transfecting BAC DNA into BHK-21 cells with Fugene-6 (Roche Diagnostics Ltd.). GFP^+^ viruses (retaining the BAC cassette) were grown directly in BHK-21 cells. To remove the BAC cassette, viruses were first passaged through NIH 3T3-CRE cells until GFP^+^ cells were no longer visible [4].

### Cell lines

MEF-1 cells (American Type Culture Collection CRL-2214), 293T cells, BHK-21 cells, NIH-3T3-CRE cells [16] 49100.2 T cells [33], and murine embryonic fibroblasts (MEFs) were grown in Dulbecco's modified Eagle medium (Invitrogen Corporation) supplemented with 2 mM glutamine, 100 U/ml penicillin, 100 µg/ml streptomycin and 10% fetal calf serum (PAA laboratories). Medium for MEFs was further supplemented with 50 µM 2-mercaptoethanol. TET-ON/OFF cells were generated by retroviral transduction. 293T cells were co-transfected with either pREV-TRE-tTA plus the retroviral packaging plasmid pEQPAM3 [34], or pMSCV-tTS-IRES-PURO plus pEQPAM3. Retroviral supernatants were harvested at 48 h and 72 h post-transfection and used to transduce MEF-1 cells in the presence of 10 µg/ml hexadimethrine bromide. Transduced cells were selected with hygromycin (250 µg/ml) and puromycin (10 µg/ml).

### Virus titers

Wild-type and TRE-50 virus titers were determined by plaque assay on BHK-21 cells [4]. Briefly, 10-fold virus dilutions were incubated on BHK-21 monolayers for 2 h. The monolayers were then overlaid with medium containing 0.3% carboxymethylcellulose. After 4 days, monolayers were fixed in 4% formaldehyde and stained with 0.1% toluidine blue. Plaques were then counted using a plate microscope. In preliminary experiments we established that TRE-50 viruses showed no growth defect on BHK-21 cells caompred to wild-type MuHV-4, and that TRE-50 virus titers on BHK-21 cells were equivalent to those on doxycycline-treated TET-ON/OFF cells (as the TRE promoter is leaky unless suppressed).

### DNA analysis

Viral DNA was isolated from infected BHK-21 cells by alkaline lysis [4], then digested with either *Bam*HI, *Bgl*I or *Eco*RI, electrophoresed on 0.8% agarose gels and transferred to positively charged nylon membranes (Roche Diagnostics Ltd.). A^ 32^P-dCTP (APBiotech) labelled probe was generated by random primer extension (Nonaprimer kit, Qbiogene) from a 4048bp *BamH*I genomic fragment (co-ordinates 64765–68813). Membranes were hybridised with probe (65°C, 18 h), washed to a stringency of 0.2% SSC, 0.1% SDS at 65°C and exposed to X-ray film. For viral DNA quantitation, samples were digested with *Pst*I and probed with a ^32^P-dCTP labelled *Pst*I genomic fragment corresponding to the MuHV-4 terminal repeat [2]. Circular MuHV-4 genomes were identified by *in situ* cell lysis and resolution in vertical gels [35]: cells were washed, pelleted and resuspended in 89 mM Tris borate, 2 mM EDTA, 15% Ficoll, 10 µg/ml RNase A, 0·01% bromophenol blue, then loaded into a 0.8% agarose gel and overlaid with an equal volume of 5% Ficoll, 1% SDS, 100 µg/ml proteinase K, 0·05% xylene cyanol green. Samples were electophoresed at 12 V for 4 h and then overnight at 80 V. The DNA was blotted onto positively charged nylon membranes and probed with the ^32^P-dCTP-labelled *Pst*I terminal repeat fragment. Membranes were washed and exposed to X-ray film as above. Viral genomes were also quantitated by real-time PCR of genomic co-ordinates 4166–4252 [36]. The PCR products were quantitated by hybridization with a Taqman probe (genomic coordinates 4218–4189) and converted to genome copies by comparison with a standard curve of cloned plasmid template amplified in parallel. Cellular DNA was quantitated in parallel by amplifying part of the adenosine phosphoribosyl transferase gene (forward primer 5′-GGGGCAAAACCAAAAAAGGA, reverse primer 5′-TGTGTGTGGGGCCTGAGTC, probe 5′-TGCCTAAACACAAGCATCCCTACCTCAA).

### RNA analysis

RNA was extracted from MuHV-4-infected BHK-21 cells with RNAzol B (Tel-Test). For Northern blots, total RNA was electrophoresed (5 µg/lane) on 1% formaldehyde agarose gels and blotted overnight onto positively-charged nylon membranes. Probes for β-actin, ORF50 and M7 were generated from PCR-amplified templates by random primer extension (Qbiogene) with ^32^P-dCTP (APBiotech). Blots were hybridized with probe overnight at 65°C, then washed (0.2xSSC, 0.1%SDS, 65°C) and exposed to X-ray film. For cDNA synthesis, any contaminating DNA was first removed by digestion with RNase-free DNase (Promega Corporation). cDNA synthesis with AMV reverse transcriptase (Promega Corporation) was then primed with oligo-dT and cDNA samples amplified by real-time PCR (Rotor Gene 3000, Corbett Research). PCR products were quantitated with Sybr green (Invitrogen Corporation) and compared with dilutions of cloned plasmid template amplified in parallel. Amplified products were distinguished from paired primers by melting curves, and the correct size of the amplified products confirmed by agarose gel electrophoresis and staining with ethidium bromide. The primers used were ORF73: 5′-TGTGCCAGAAGCTTGTGTA, 5′- ATATCAGGGAATGCGAAGAC; ORF25: 5′-ATCGCCTGTCTCAATACTGAATTCAA, 5′-GAAGAAGGTGTGCTCTAGTAGATGC; K3: 5′-TCTTTGTGGGCTGCTGGGT, 5′- TGGCTGTGCTGATGATAGTGATG; ORF50: 5′-ATCATTAACCTGGACCCT, 5′- TAAGCCTGTTCGTGCCCAGAAG; ORF46: 5′-TTGCCTTGTTTCCCCACAGCATAAA, 5′-GTCAGGATGCAGTTAAGCAGAAGAA.

### Immunoblotting

Cells were lysed on ice for 30 minutes in 50 mM TrisCl pH 7.4, 150 mM NaCl, 5 mM EDTA, 1% Triton X-100, 1 mM PMSF. Nuclei and debris were pelleted by centrifugation (13,000×*g*, 15 min). Supernatants were mixed 1∶1 with 2× Laemmli's loading buffer, resolved by PAGE, then transferred to PVDF membranes (Perbio Science). Membranes were blocked with PBS/0.1% Tween-20/10% non-fat milk, and probed with a MuHV-4-immune rabbit serum [32], followed by horseradish peroxidase-conjugated donkey anti-rabbit pAb (Dako-Cytomation). The immunoblots were then developed with ECL reagents (APBiotech) and exposed to X-ray film.

### Antigen presentation assay

TRE-50 virus was reactivated from TET-ON/OFF cells (5×10^4^/well) with doxycycline, then incubated (18 h, 37°C) with 49100.2 T cells (10^5^/well) [33], which are specific for the H2-D^b^-restricted p56 epitope of MuHV-4 (AGPHNDMEI) [20], and produce beta-galactosidase in response to T cell receptor signalling [37]. To assay beta-galactosidase production, the cells were washed in PBS and lysed in PBS with 5 mM MgCl_2_, 1% NP-40, 0.15 µM chlorophenol-red-beta-D-galactoside (Merck Biosciences). After 4–6 h the absorbance at 595 nm was read on a Biorad Benchmark Microplate Reader.

### Flow cytometry

Cells infected with GFP^+^ viruses were washed (0.1% BSA, 0.01% azide in PBS) and analysed directly for green channel fluorescence. For specific staining, cells were incubated with rabbit anti-MuHV-4 immune serum [11] (1 h, 4°C), washed x2 in PBS, incubated with fluorescein-conjugated pig anti-rabbit pAb (Dako Cytomation) (1 h, 4°C), washed x2 in PBS and analysed on a FACS Calibur using Cellquest software (BD Biosciences).

### Immunofluorescence

Cells were fixed in 4% formaldehyde (30 min), then permeabilized with 0.1% Triton-X100 +0.1% Tween-20 in PBS. The MuHV-4 ORF65 capsid component was identified by staining with mAb MG-12B8 [38] plus Alexa-568-conjugated goat-anti-mouse IgG pAb. EGFP was visualized directly. Nuclei were counter-stained with DAPI. Staining was visualized with an Olympus IX70 microscope plus a Retiga 2000R camera line (QImaging).

## Results

### Establishment of cell lines with latent, reactivatable MuHV-4

**Figure 1:**
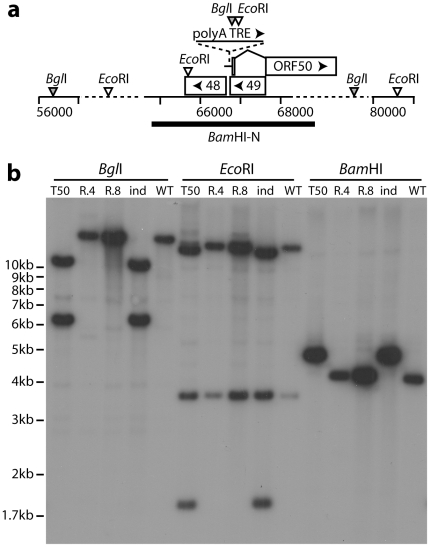
Modification of MuHV-4 ORF50 control. **a**. Schematic diagram showing the insertion in the 5′ untranslated region of ORF50, between MuHV-4 ORFs 48 and 49, of a poly-adenylation site plus a downstream promoter (TRE) with binding sites for doxycycline-dependent transcription regulators. **b**. Viral DNA from the TRE-50 mutant (T50), an independent mutant (ind), a revertant of each mutant (R.4, R.8) and wild-type MuHV-4 (WT) was digested with *Bgl*I, *Eco*RI or *Bam*HI, electrophoresed and probed with a *Bam*HI-N fragment as shown in **a**. The polyA-TRE insertion (705bp) introduces a *Bgl*I site, such that a wild-type 16476bp band becomes 11040bp + 6141bp, and an *Eco*RI site, such that a 14937bp wild-type band becomes 13926bp + 1716bp. The 3525bp *Eco*RI band is unchanged. The insertion makes the 4048bp wild-type *Bam*HI band 4753bp.

We previously de-regulated the MuHV-4 lytic cycle by inserting an additional promoter element in the 5′ untranslated region of ORF50 [39], a gene both necessary and sufficient to drive lytic replication [12], [40]. We used a similar strategy here to down-regulate ORF50, adding an SV40 polyadenylation signal to its 5′ untranslated region. We also added a doxycycline-inducible promoter downstream of the polyadenylation signal (Fig. 1a). Thus, endogenous ORF50 transcripts were replaced with doxycycline-inducible transcripts. Southern blots confirmed the predicted genomic structures of the recombinant (TRE-50) viruses (Fig. 1b). We next generated a cell line (TET-ON/OFF) with a doxycycline-inactivated, TRE-binding transcriptional suppressor and a doxycycline-activated, TRE-binding transcriptional activator. The suppressor was expressed constitutively from an HCMV IE1 promoter and the transactivator from a doxycyline-inducible promoter, such that doxycycline induced ORF50 expression but without doxycycline ORF50 transcription was actively suppressed.

**Figure 2:**
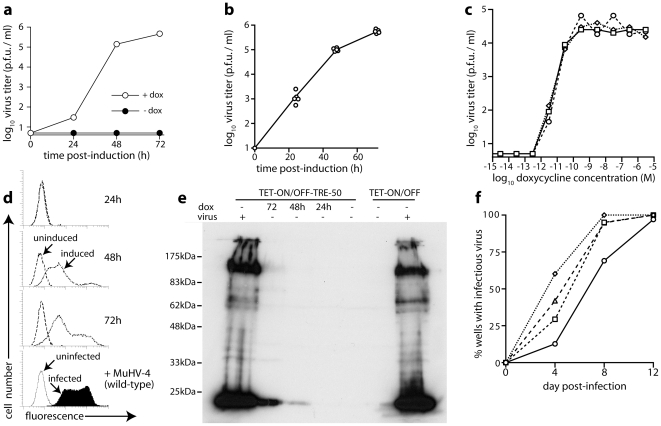
Reactivation of TRE-50 MuHV-4 from latently infected fibroblasts. **a**. TET-ON/OFF cells were infected with TRE-50 MuHV-4 (3 p.f.u. /cell) then cloned. Cultures of a representative clone were analyzed for infectious virus by plaque assay after induction with doxycycline (dox, 1 µg/ml). Infectious virus was undetectable without induction. **b**. Plaque titers after doxycycline treatment were determined for an additional 5 clones. The x axis marks the lower limit of assay sensitivity. Again no infectivity was detected without induction. **c**. 3 clones were tested for sensivity to doxycycline. Virus titers were determined by plaque assay 48 h after adding doxycycline at the final concentration shown. **d**. A latently infected TET-ON/OFF cell clone was induced with doxycycline for the time indicated (dotted lines) or left uninduced (dashed lines), then stained with a MuHV-4-immune rabbit serum and analyzed by flow cytometry. In the bottom panel, the cells were infected with wild-type MuHV-4 without doxycycline (filled histogram) or left uninfected. **e**. A latently infected clone (TET-ON/OFF-TRE-50) or uninfected cells (TET-ON/OFF) were treated or not with doxycycline for the time indicated, then analyzed for MuHV-4 antigen expression by immunoblotting with a MuHV-4-immune rabbit serum. As a positive control, wild-type MuHV-4 (virus +) was added 18 h before analysis. **f**. Latently infected TET-ON/OFF cell clones were sub-cloned further and then cultured with doxycycline. Sub-clone supernatants were analyzed for infectious virus by incubation with BHK-21 cells for 4 days and then scoring or not for cytopathic effects. Each graph shows the percentage of subclones yielding infectious virus with time. At least 70 sub-clones were analyzed for each latently infected clone.

We infected these cells and cloned them cells 4 h later to establish uniform, latently infected populations. The minority of clones showing cytopathic effects were discarded. Infectious virus remained undetectable in supernatants of most clones over at least 4 weeks, but reappeared 24–48 hours after exposure to doxycycline. Fig. 2a shows a typical example; Fig. 2b shows 5 further clones with similar patterns of latency and doxycycline-induced reactivation. Thus, we could reproducibly establish fibroblast clones that carried a replication-competent viral genome and were fully permissive for MuHV-4 lytic replication, but were latently infected. Fig. 2c shows a titration of doxycycline dose for such 3 clones.

MuHV-4 lytic antigens were undetectable in uninduced clones and abundant 14 h after exposure to exogenous, wild-type virions (Fig. 2d–e). Lytic antigen expression was also evident after treatment with doxycycline to reactivate endogenous viral genomes. However, its onset was slow, being undetectable after 24 h and weak even after 72 h, compared to the 14 h exogenous infection. This suggested that while latent virus could be consistently reactivated, this might be occuring in only a minority of the doxycycline-treated cells. To establish that the cultured cells had not lost their viral genomes, we subcloned them just before doxycycline treatment and then scored each subclone for infectious virus production (Fig. 2f). Reactivation was again slow, but essentially all the subclones of each latently infected clone yielded detectable infectious virus, indicating that they maintained at least one reactivation-competent viral genome.

### Direct visualization of latent infection

**Figure 3:**
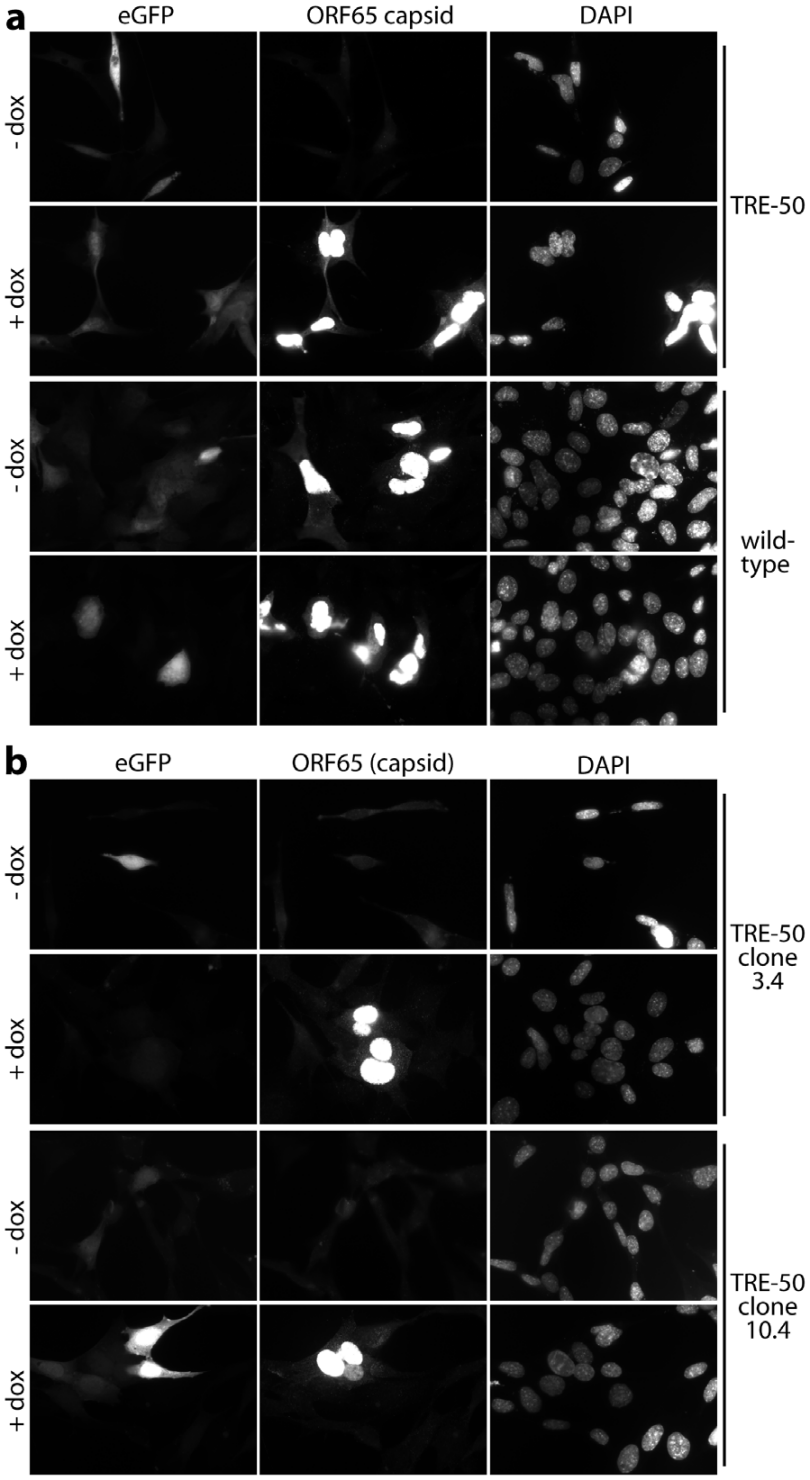
EGFP and virion capsid expression by latently infected and reactivating cells. **a**. TET-ON/OFF cells were infected (0.1 p.f.u. /cell) with wild-type eGFP^+^ or TRE-50 eGFP^+^ MuHV-4, then treated or not with doxycycline (dox, 1 µg/ml). 48 later the cells were fixed, permeabilized and stained for ORF65 capsid expression with mAb MG-12B8. EGFP expression was visualized directly. Nuclei were counterstained with DAPI. **b**. Latent eGFP^+^ TRE-50 clones were established and reactivation then induced or not with doxycycline. 48 h later the cells were fixed, permeabilized and stained as in **a**. 2 representative clones (3.4, 10.4) are shown.

BAC-derived MuHV-4 that retains the BAC cassette expresses eGFP from an HCMV IE1 promoter at the left end of the viral genome [32]. This promoter operates independently of other viral genes: in either lytic infection or in latency it can be either on or off [9], [10]. Thus it marks a subset of latently infected cells. TET-ON/OFF cells infected with wild-type eGFP^+^ MuHV-4 showed eGFP and capsid expression regardless of doxycycline treatment (Fig. 3a). TET-ON/OFF cells infected at the same multiplicity with TRE-50 eGFP^+^ MuHV-4 showed eGFP expression without capsid expression. When the cells were treated with doxycycline, eGFP and capsid expression were both evident (although not always in the same cells). Established, latently infected clones also showed eGFP expression without detectable capsids (Fig. 3b), although the proportions of eGFP^+^ cells were low, suggesting that the HCMV IE1 promoter tended to become silenced.

### Analysis of latently infected cells

**Figure 4:**
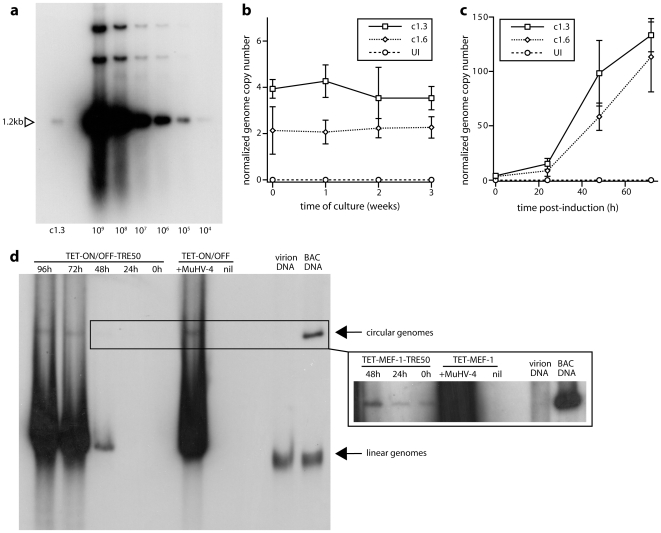
Analysis of latently infected TET-ON/OFF cells. **a**. A latently infected TET-ON/OFF cell clone (c1.3) was analyzed for genome copy number by Southern blot (100 ng DNA) for the viral 1.2 kb *Pst*I terminal repeat fragment. For comparison we titrated purified MuHV-4 BAC DNA-estimated viral genome copy numbers are shown. 100 ng DNA is equivalent to approximately 10^4^ cells. Therefore by this method c1.3 contained 1–3 viral genomes per cell. **b**. The viral genome copy number of 2 latently infected TET-ON/OFF cell clones (c1.3, c1.6) was determined by real-time PCR of 10 ng DNA. Values were normalized by the cellular APRT copy number of each sample to give the number of viral genomes per 2 copies of host DNA, i.e. per cell. Each point shows mean ± SD of triplicate samples. No change in copy number was observed over 3 weeks of continuous culture. UI = uninfected TET-ON/OFF cells. **c**. The viral genome copy numbers per 2 copies of cellular APRT were determined for c1.3 and c1.6 following doxycycline treatment (1 µg/ml). Each point shows mean ± SD of triplicate samples. UI = uninfected cells. **d**. c1.3 cells (TET-ON/OFF-TRE-50) were treated with doxycycline for the time indicated (0 h = no doxycycline), then lysed *in situ*. Circular and linear viral genomes were separated by electrophoresis, and Southern blots probed for the viral 1.2 kb terminal repeat. Controls included virion DNA (linear), BAC-cloned viral DNA (circular, although some has sheared to become linear) and TET-ON/OFF cells either uninfected (nil) or exposed overnight to wild-type MuHV-4 (+MuHV-4, mostly linear). The inset shows a longer exposure to reveal low copy number circular viral genomes.

Southern blots for the viral terminal repeat unit (Fig. 4a) established that latently infected fibroblasts contained on average <5 MuHV-4 genomes per cell. (Fig. 2f shows that such populations did not include a significant number of genome-negative cells.) This result was confirmed by quantitative PCR: 6 independent clones contained 3.2±1.3 viral genomes/cell (mean±SD). Genome copy numbers remained stable over at least 3 weeks of culture without induction (Fig. 4b). Doxycycline treatment (Fig. 4c) then increased genome copy numbers at a rate consistent with the earlier infectivity assays. Gardella gel analysis (Fig. 4d) of TET-ON/OFF-TRE50 cells was difficult because of the low viral genome copy numbers, but nonetheless showed circular genomes in uninduced cells, consistent with stable latency rather than an abortive infection.

### Transcriptional analysis of reactivating viral genomes

**Figure 5:**
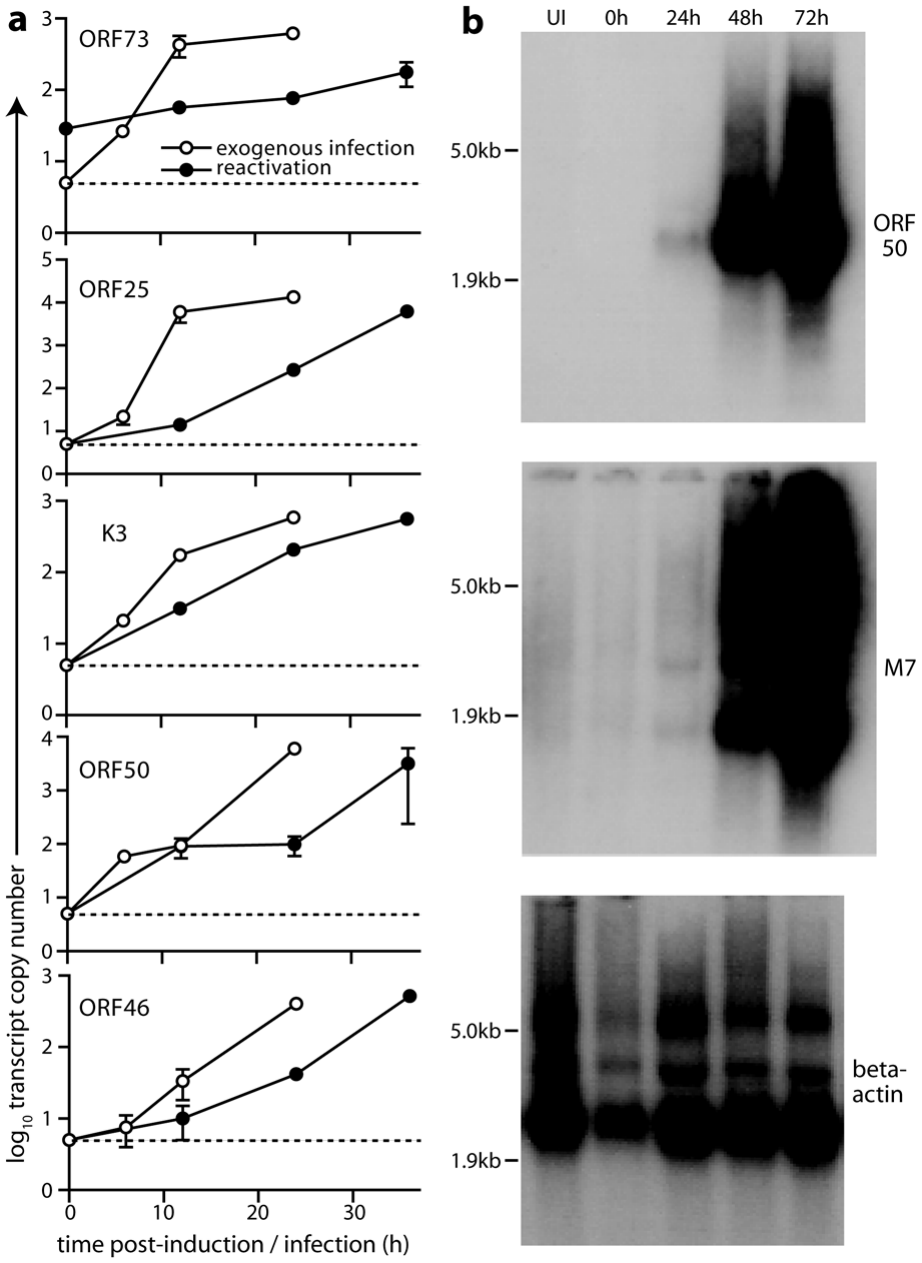
Transcriptional changes associated with MuHV-4 reactivation. **a**. Latently infected TET-ON/OFF cells were analyzed for MuHV-4 transcripts by RT-PCR before and after treatment with doxycycline (filled symbols). As a control, uninfected TET-ON/OFF cells were exposed to wild-type MuHV-4 (open symbols). Each point shows the mean ± SD copy number of triplicate RT-PCR reactions, adjusted to give the number of viral transcripts per 10^3^ beta-actin transcripts in the same sample. Reverse transcriptase-negative controls always gave viral mRNA copy numbers that were undetectable or <1% of reverse transcriptase-positive samples. **b**. RNA (5 µg) was extracted from c1.3 cells either before induction (0 h) or after induction with doxycycline (1 µg/ml). UI = uninfected TET-ON/OFF cells. Replicate blots were probed for ORF50, M7 (a MuHV-4 late gene) or beta-actin.

We used RT-PCR to analyze MuHV-4 transcription in latently infected and reactivating fibroblasts. mRNA was extracted from TET-ON/OFF-TRE50 cells after doxycycline treatment, and compared with mRNA extracted from TET-ON/OFF cells either uninfected or exposed to wild-type virions (Fig. 5a). mRNA for the ORF73 episome maintenance protein was present during both latency and reactivation. ORF50 mRNA was not detected in latency, but was readily detected after induction. K3 mRNA was similarly present only after induction, consistent with the ORF50 dependence of its promoter [41]. ORF46 (an early gene) and ORF25 (a late gene) [42] showed similar induction kinetics to ORF50. Northern blots (Fig. 5b) comparing ORF50 and a late gene (M7) again gave a similar picture. Thus, there was no sign of a much more rapid induction of immediate early/early genes than late genes that might have indicated slow progress through the lytic cycle after induction-all lytic transcripts were undetectable without induction, sparse at 24 h, and abundant at 48–72 h.

### Protection of reactivating virus by K3

**Figure 6:**
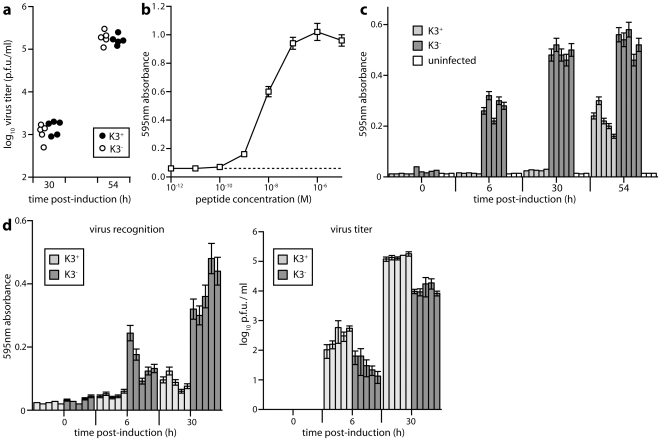
Antigen presentation by reactivating MuHV-4. **a**. TET-ON/OFF cells latently infected with K3^+^ or K3^−^ TRE-50 MuHV-4 were treated with doxycyline (1 µg/ml), and virus titers determined by plaque assay 30 or 54 h later. Each point shows a separate clone. Infectious virus was undetectable without induction. **b**. Uninfected TET-ON/OFF cells were incubated with different doses of p56 peptide (2 h, 37°C), then washed x2 in DMEM and used to stimulate the MuHV-4-specific T cell hybridoma 49100.2 (16 h, 37°C). Beta-galactosidase production by the hybridoma cells was assayed by adding CPRG to cell lysates and reading the absorbance at 595 nm. Bars show mean ± SD of 3 replicate cultures. **c**. TET-ON/OFF clones either uninfected or latently infected with K3^+^ or K3^−^ TRE-50 MuHV-4 were incubated with doxycycline + phosphonoacetic acid (100 µg/ml) for the time incubated, then for a further 16 h with 49100.2 cells before assaying beta-galactosidase production as in **b**. Each bar shows mean ± SD of 3 replicate cultures for 1 clone. Hybridoma stimulation was significantly greater by reactivation K3^−^ MuHV-4 than by K3^+^ at all time points after induction (p<0.0001 by Student's 2-tailed t test). **d**. TET/ON-OFF cell clones carrying latent K3^+^ or K3^−^ TRE-50 MuHV-4 were induced with doxycycline without phosphonoacetic acid for the times indicated. MuHV-4-specific 49100.2 T cells were then added to each culture. Replicate cultures were assayed 16 h later for viral antigen recognition by beta-galactosidase assay, or for virus titer by plaque assay. Bars show mean ± SD of triplicate cultures for separate clones. K3^−^ clones were both recognized significantly better by 49100.2 cells and significantly inhibited in virus production compared to K3^+^ clones (p<0.01 by Student's 2-tailed t test).

To test whether CD8^+^ T cell evasion contributes to MuHV-4 reactivation, we disrupted its K3 evasion gene on the TRE-50 background and derived K3^−^ and K3^+^ latently infected cells. K3^−^ and K3^+^ viruses showed no obvious difference in their capacity to establish latently infected clones. Average viral genome copy numbers (mean±SD, 5 clones each) were also indistinguishable - 3.0±1.2 for K3^+^ and 3.2±1.3 for K3^−^-as were virus titers after induction (Fig. 6a).

We tested viral antigen presentation using the 49100.2 T cell hybridoma, which recognizes an H2-D^b^-restricted epitope of ORF6, a lytic gene. Fig. 6b shows the relationship between antigen dose and hybridoma response. Reactivating K3^−^ clones, treated with phosphonoacetic acid to prevent any post-induction spread of lytic infection, showed significantly better epitope presentation than K3^+^ (Fig. 6c). The 49100.2 hybridoma can also exert anti-viral effects. Without phosphonoacetic acid treatment, K3^−^ clones showed both better antigen presentation and lower virus titers when 49100.2 cells were added (Fig. 6d). Therefore K3 protected the reactivating cells against CD8^+^ T cell recognition.

## Discussion

We controlled MuHV-4 reactivation by manipulating ORF50 transcription. This confirmed a central role for ORF50 in reactivation from latency and allowed us to compare single gene knockout viruses for a reactivation phenotype. Thus, K3 was shown to be important for reactivating fibroblasts to evade CD8^+^ T cell recognition.

The accumulation of lytic antigens and infectious virus in latently infected fibroblast populations was notably slower after doxycycline-induced reactivation than after the initiation of lytic infection by exogenous virions. Since the induction of early and late viral lytic transcripts was not significantly delayed compared to that of ORF50, it appeared that the initiation of reactivation was delayed rather than progress through the lytic cycle itself. Thus, viral gene expression increased with time due to more genomes reactivating and to some lytic spread from cells in which reactivation had already occured. The failure of all latent genomes to respond synchronously to doxycycline presumably reflected silencing, either of the TRE promoter in host genome, such that a doxycycline-dependent transactivator was not produced, or of the TRE promoter in the viral genome, such that the transactivator did not function. Silencing has been observed both for endogenous gammaherpesvirus promoters [43]–[45] and for TRE promoters in a non-viral context [46]. It correlates with CpG methylation and histone deacetylation [47], [48]. Methylation of the ORF50 promoter has been reported for MuHV-4 [49]; another study found a more important role for histone deacetylation [45]. Methylation can be reversed with 5-azacytidine, and histone acetylation with sodium butyrate or Trichostatin A. However, none of these treatments markedly upregulated doxycycline-dependent or doxycycline-independent TRE-50 virus reactivation; nor was delivering the Herpes simplex virus ICP0 via a recombinant adenovirus vector effective (data not shown). Therefore the mechanism of silencing remained unclear. High level reactivation ablates MuHV-4 persistence *in vivo* [39], so gammaherpesviruses have presumably evolved multiple levels of regulation to guard against this. It is also possible that ORF50 expression alone reactivates MuHV-4 less efficiently than physiological triggers, which might affect the viral genome in multiple ways.

While the efficiency of doxycycline-induced MuHV-4 reactivation was far from 100%, it was nonetheless sufficient to establish a reactivation phenotype for K3 deficiency, something not previously possible with pathogenesis assays [16]. *In vivo* MuHV-4 reactivation presumably starts with a latently infected B cell differentiating in a mucosal or sub-mucosal site, as proposed for EBV [50]. Thus, extending the current reactivation system to B cells is a priority. However, gamma-2-herpesviruses may also undertake further, local rounds of latency establishment and reactivation, as reflected in the persistence of MuHV-4 infection in B cell-deficient mice. Therefore latency and reactivation is non-B cells is also likely to be an important feature of the viral lifecycle. In contrast to the frequent KSHV genome loss observed from Kaposi's Sarcoma spindle cells [51], MuHV-4 episomes were maintained in transformed fibroblasts for at least 3 weeks without evidence of viral lytic gene expression. This would correspond to approximately 50 cell divisions, so even a 5% loss rate should have reduced a 100% genome^+^ population to <10% genome^+^. Viral episomes entirely lacking ORF50 [36] are also well maintained in fibroblasts (data not shown). Therefore our *in vitro* data support the idea of stromal cells being a possible site of long-term MuHV-4 persistence.

Isolated reactivating cells presumably rely on K3 to protect them directly against CD8^+^ T cell recognition. Once infection spreads, trans-acting evasion by the M3 chemokine binding protein [52], [53] may also become important, with K3 acting indirectly by protecting the cells that secrete M3 [54]. Thus, multiple roles for K3 are possible. The system described here provides a basis for further investigation and identifies K3 as an important gene for productive reactivation in the face of virus-specific CD8^+^ T cells.
